# Stromal Expression of MARCKS Protein in Ovarian Carcinomas Has Unfavorable Prognostic Value

**DOI:** 10.3390/ijms19010041

**Published:** 2017-12-23

**Authors:** Raoudha Doghri, Maroua Manai, Pascal Finetti, Maha Driss, Emilie Agavnian, Marc Lopez, Meriam Elghardallou, Emmanuelle Charafe-Jauffret, Mohamed Manai, Max Chaffanet, Daniel Birnbaum, Karima Mrad, François Bertucci

**Affiliations:** 1 Département d’Anatomie Pathologique, Institut Salah Azaiez, Bab Saadoun, Tunis 1006, Tunisia; raoudha.doghri@gmail.com (R.D.); maroua.m@hotmail.com (M.M.); mdriss808@gmail.com (M.D.); karimamrad@ymail.com (K.M.); 2 Département d’Oncologie Moléculaire, Centre de Recherche en Cancérologie de Marseille, Institut Paoli-Calmettes, INSERM UMR1068, CNRS UMR7258, Aix-Marseille Université, 13007 Marseille, France; finettip@ipc.unicancer.fr (P.F.); marc.lopez@inserm.fr (M.L.); chaffanetm@ipc.unicancer.fr (M.C.); daniel.birnbaum@inserm.fr (D.B.); 3 Département de Biologie, Unité de Biochimie et Biologie Moléculaire, Faculté des Sciences de Tunis, Université de Tunis El Manar, Tunis 1068, Tunisia; mohamed.manai@fst.rnu.tn; 4 Département de Bio-Pathologie, Institut Paoli-Calmettes, 13009 Marseille, France; agavniane@ipc.unicancer.fr (E.A.); jauffrete@ipc.unicancer.fr (E.C.-J.); 5 Département de Médicine Communautaire, Faculté de Médecine de Sousse, Sousse 4000, Tunisia; meriamelghardallou@yahoo.fr; 6 UFR de Médecine, Aix Marseille Université, 13007 Marseille, France; 7 Département d’Oncologie Médicale, Institut Paoli-Calmettes, 13009 Marseille, France

**Keywords:** epithelial ovarian cancer, immunohistochemistry, MARCKS, prognosis, stroma, survival

## Abstract

Epithelial ovarian cancer (EOC) is the most lethal gynecological cancer. Identification of new therapeutic targets is crucial. MARCKS, myristoylated alanine-rich C-kinase substrate, has been implicated in aggressiveness of several cancers and MARCKS inhibitors are in development. Using immunohistochemistry (IHC), we retrospectively assessed MARCKS expression in epithelial and stromal cells of 118 pre-chemotherapy EOC samples and 40 normal ovarian samples from patients treated at Salah Azaiez Institute. We compared MARCKS expression in normal versus cancer samples, and searched for correlations with clinicopathological features, including overall survival (OS). Seventy-five percent of normal samples showed positive epithelial MARCKS staining versus 50% of tumor samples (*p* = 6.02 × 10^−3^). By contrast, stromal MARCKS expression was more frequent in tumor samples (77%) than in normal samples (22%; *p* = 1.41 × 10^−9^). There was no correlation between epithelial and stromal IHC MARCKS statutes and prognostic clinicopathological features. Stromal MARCKS expression was correlated with shorter poor OS in uni- and multivariate analyses. Stromal MARCKS overexpression in tumors might contribute to cancer-associated fibroblasts activation and to the poor prognosis of EOC, suggesting a potential therapeutic interest of MARCKS inhibition for targeting the cooperative tumor stroma.

## 1. Introduction

Epithelial ovarian cancer (EOC) is the most lethal gynecological cancer because of often late diagnosis and high recurrence rate [1]. Despite initial chemosensitivity, ovarian cancer is not chemocurable. One reason of the high mortality rate is the lack of effective therapeutic options in case of chemoresistance. According to the Cancer Registry of Northern Tunisia, EOC is the second gynecological cancer with an incidence rate of 3.9 per 100,000 inhabitants [2]. It is distributed into four major pathological types: “serous” (75%), “endometrioid” (10%), “mucinous” (3%), and “clear cell” (10%), which differ in pathogenesis, molecular alterations, and prognosis [3,4]. The classical clinicopathological prognostic factors include patient's age, Fédération Internationale de Gynécologie et d Obstétrique (FIGO) stage, pathological tumor grade and type, and initial surgery results. Optimal debulking surgery and paclitaxel/platinum-based chemotherapy have improved survival [5,6,7]. Nevertheless, the overall 5-year survival rate for patients with EOC is still very low, approximately 30% [8,9]. In Tunisia, overall survival is 27% at 5-years for advanced-stage EOC [10]. Several molecular subtypes of EOC [11,12] and prognostic gene expression signatures [13,14] have been reported, but without clinical application to date.

MARCKS, myristoylated alanine-rich C-kinase substrate, a substrate for protein kinase C, is localized in the plasma membrane and is an actin filament cross-linking protein. Phosphorylation by protein kinase C or binding to calcium-calmodulin inhibits its association with actin and plasma membrane, leading to its presence in the cytoplasm. MARCKS plays an important role in the regulation of cytoskeletal plasticity and especially of actin filaments [15,16]. Ubiquitously expressed in various tissues [17], it is involved in cell motility, cell adhesion, cytokines secretion [18] phagocytosis, membrane trafficking [19], and mitogenesis [20]. Many studies have shown the implication of MARCKS in cancer aggressiveness, notably metastatic process and therapeutic resistance [21,22,23,24,25,26]. A few studies also demonstrated the efficiency of therapeutic inhibition of MARCKS [27,28]. A single study of MARCKS expression in EOC has shown that MARCKS is highly expressed in the ovarian stroma, is required for the differentiation and tumor-promoting function of cancer-associated fibroblasts (CAFs) and is associated with poor survival [26].

We have here analyzed MARCKS protein expression in stromal and epithelial cells in tumor samples of patients with EOC treated at Salah Azaiez Institute of Tunis, Tunisia, and searched for correlations with clinicopathological features and survival. We found more frequent stromal MARCKS expression in tumor samples (77%) than in normal samples (22%), and correlation with shorter overall survival in uni- and multivariate analyses, suggesting that MARCKS inhibition might represent a new therapeutic approach in EOC.

## 2. Results

### 2.1. Patients’ Population and Clinicopathological Features

The clinicopathological features of all samples (*N* = 118) are shown in Table 1. The patients had a median age of 55 years at diagnosis (range, 27–85). As expected, most of the patients (91%) had an advanced stage III–VI (FIGO), including 21 cases (18%) with metastasis at diagnosis. The most frequent pathological type was serous (90%), followed by clear cell carcinoma (4%), mixed (3.5%) and endometrioid (2.5%). Most of the cases were high-grade (grade 3: 70%). All cases were operated before or after chemotherapy, with a macroscopic tumor residue in 35% of cases. Adjuvant and/or neoadjuvant chemotherapy was delivered to 89 patients.

### 2.2. MARCKS Protein Expression in Epithelial Ovarian Cancer

We first validated the MARCKS antibody using western blot analysis on three breast cancer cell lines with known *MARCKS* mRNA expression. As shown in Figure S1, the antibody specifically recognized MARCKS protein with a good correlation between protein and mRNA expression levels.

**Figure 1:**
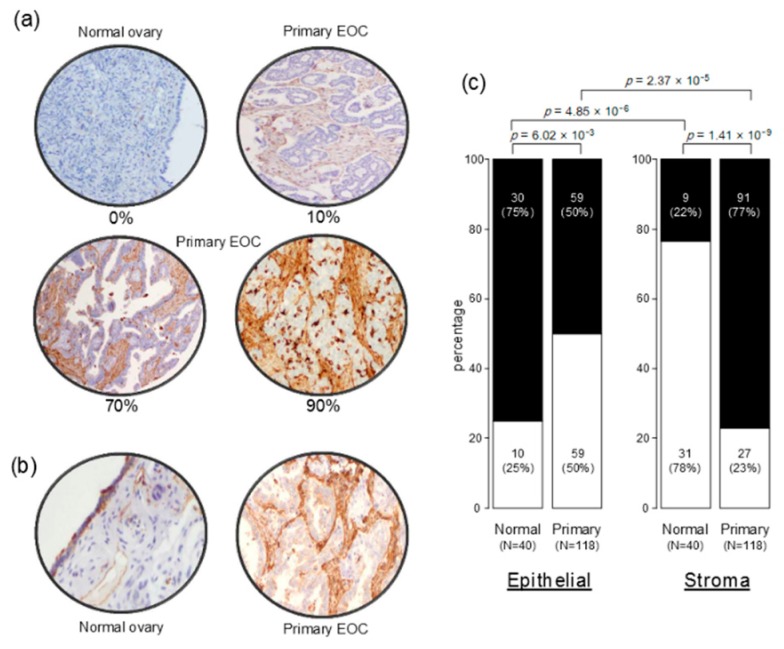
MARCKS (myristoylated alanine-rich C-kinase substrate) immunostaining in epithelial ovarian cancer and normal ovarian samples. (**a**) Representative images (×200) of stromal MARCKS immunohistochemistry (IHC) staining in normal ovary (left top panel: 0% positive cells), and in three primary epithelial ovarian cancer (EOC) samples showing different percentages of stained cells score: 10%, 70%, and 90%; (**b**) Representative images (×200) of normal ovary with weak expression of MARCKS in stromal cells but strong expression in epithelial cells (**left**) and of primary EOC sample with strong expression in stromal fibroblasts and weak expression in epithelial tumor cells (**right**); (**c**) Box plots showing the percentage of MARCKS-positive (black) samples and MARCKS-negative (white) samples (normal, primary tumors) for the epithelial staining (**left**) and stromal staining (**right**). The *p*-values are for the Fischer’s exact test.

MARCKS protein expression was then measured on the 118 tumor samples and 40 normal samples present on the tissue-microarray (TMA). Examples of staining are shown in Figure 1a and results are summarized in Figure 1b. Using 1% of stained epithelial cells as positivity cut-off, we found that 75% of normal samples showed positive MARCKS immunostaining versus 50% of tumor samples (*p* = 6.02 × 10^−3^; Fisher’s exact test). Regarding the stroma staining and using the same positivity cut-off (1% of stained stromal cells), 77% of tumor samples showed positive MARCKS immunostaining versus 22% of normal samples (*p* = 1.41 × 10^−9^; Fisher’s exact test). In tumor samples, the staining was observed mainly in stromal cells, notably fibroblasts (77% positivity) and to a lesser degree in tumor epithelial cells (50% positivity; *p* = 2.37 × 10^−5^, Fisher’s exact test), whereas in normal tissues, it was weakly expressed in stromal cells (22% positivity) and more expressed in epithelial cells (75% positivity; *p* = 4.85 × 10^−6^, Fisher’s exact test).

### 2.3. Correlation of MARCKS Protein Expression with Clinicopathological Features

We analyzed correlations between the binary MARCKS IHC status and prognostic clinicopathological features of samples including patients’ age, FIGO stage, pathological type and grade, and macroscopic tumor residue after debulking surgery (Table 2). We did not find any correlation between epithelial MARCKS expression and clinicopathological features. Similarly, the stromal IHC status did not correlate with any tested feature. Epithelial and stromal IHC MARCKS status were not correlated.

### 2.4. Correlation of MARCKS Protein Expression with Overall Survival

**Figure 2:**
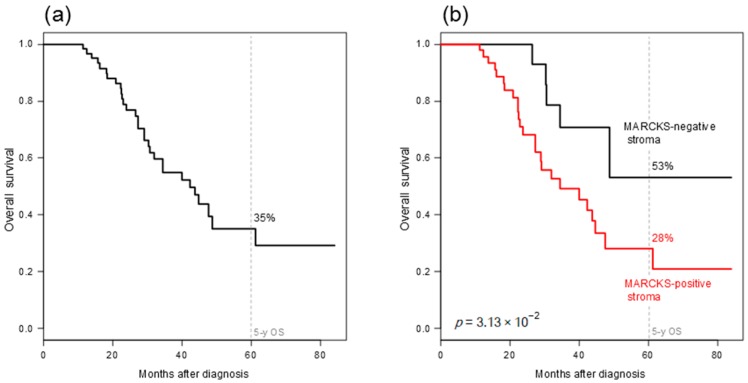
Overall survival in patients with epithelial ovarian cancer. (**a**) Kaplan-Meier OS curve in 68 patients treated with treated with surgery and carboplatin-paclitaxel chemotherapy and with available follow-up; (**b**) Similar to (**a**), but according to the stromal IHC MARCKS status. The *p*-value of log-rank test is indicated.

We then assessed the prognostic value of MARCKS expression for OS in the population of 68 patients treated with surgery and carboplatin-paclitaxel chemotherapy and with available follow-up. Fifteen of 68 patients (22%) died and the 5-year OS was 35% [95CI 0.22–0.56] (Figure 2a).

In univariate analysis (Table 3), high grade (*p* = 2.16 × 10^−2^, Wald test; HR = 2.51 [95CI, 1.14–5.49]) and positive stromal MARCKS status (*p* = 3.77 × 10^−2^, Wald test; HR = 2.59 [95CI, 1.06–6.36]) were associated with poor OS, whereas epithelial MARCKS status was not (*p* = 0.881). In multivariate analysis (Table 3), the grade remained significant (*p* = 4.82 × 10^−2^, Wald test; HR = 2.21 [95CI, 1.01–4.86]), whereas the stromal MARCKS status tended to remain significant (*p* = 7.90 × 10^−2^, Wald test; HR = 2.24 [95CI, 0.91–5.53]).The 5-year OS rate was 28% [95CI 0.15–0.52] in the stromal MARCKS-positive group versus 53% [0.27–1] in the stromal MARCKS-negative group (*p* = 3.13 × 10^−2^, log-rank test; Figure 2b).

## 3. Discussion

Despite therapeutic progresses achieved during the last decades, the survival of patients with EOC remains poor, and the identification of new therapeutic targets is crucial. The objective of this study was to evaluate and compare MARCKS protein expression in stromal and epithelial cells in a large retrospective series of 118 EOC samples collected from Tunisian patients and to search for correlations with clinicopathological features. We have shown that MARCKS tumor expression is more frequent in stromal cells than in epithelial cells, and that stromal MARCKS expression is associated with shorter overall survival (OS).

We focused on MARCKS protein expression for several reasons: (i) proven role of MARCKS in cancer progression including metastasis and therapeutic resistance; (ii) ongoing development of MARCKS inhibitors [27,28]; (iii) commercial availability of a corresponding monoclonal antibody performing sufficiently well in IHC on paraffin-embedded tissues, as previously reported [26,29]. Before analysis of tissue samples, we revalidated the antibody on cancer cell lines by using western blot analysis.

MARCKS expression in our series of 118 EOC samples and 40 normal ovarian samples was heterogeneous in all samples. We found that 75% of normal samples showed positive epithelial staining versus 50% of tumor samples. Regarding the stroma staining and with the same positivity cut-off, 77% of tumor samples showed positive staining versus 22% of normal samples. Because secretory epithelial cells of the fallopian tubes are the precursors of high-grade serous ovarian tumors, we analyzed MARCKS expression in 27 samples of normal fallopian tube (Figure S2). Interestingly, the results were very similar to those observed in the normal ovarian samples for both epithelial (74% positivity versus 75% in normal ovarian samples) and stromal staining (19% positivity versus 22%). In tumor samples, the staining was observed mainly in stromal cells, notably fibroblasts, and to a lesser degree in tumor epithelial cells, whereas in normal tissues, it was weakly expressed in stromal cells and more expressed in epithelial cells. Thus, MARCKS protein was overexpressed in ovarian tumor stroma as compared to epithelial cells. In the literature, only one study analyzed MARCKS protein expression in epithelial ovarian cancer [26] and found similar results. From *in silico* analyses of public transcriptional data, the authors first showed that, compared with its expression in the tumor epithelial compartment, MARCKS was specifically expressed in the stromal compartment. In contrast, MARCKS level was reduced in the tumor epithelial cells compared with normal ovary epithelial. Second, using IHC analyses in a small series of 10 normal ovarian tissues and 18 pairs of primary and metastatic tumors from patients with advanced (stages III–IV) serous adenocarcinoma, they showed that MARCKS protein was highly expressed in ovarian tumor stroma versus epithelial cells compartment. Additionally, MARCKS was highly expressed in normal ovary epithelial cells compared with tumor epithelial cells. We confirmed these results in our present larger study. The authors found at the transcriptional level in a series of 3431 ovarian cancer specimens a small correlation between high stroma MARCKS expression and higher FIGO stage. In our series, there was no correlation between stromal or epithelial protein staining and the classical clinicopathological prognostic features, including the FIGO stage. Finally and despite the relatively small size of our series (68 informative cases), we found that stromal MARCKS expression in tumors was associated with shorter OS in univariate analysis (HR = 2.59), and that such correlation tended towards significance in multivariate analysis with a hazard ratio equal to 2.24. Such correlation was reported [26], but at the transcriptional level (but not at the protein level) in terms of progression-free survival and OS. It was suggested that MARCKS stromal overexpression might contribute to cancer-associated fibroblasts activation in EOC and explain their therapeutic resistance and unfavorable prognostic impact. In experimental models, MARCKS overexpression was shown to suppress cellular senescence and boost the activation of AKT/TWIST1 (Protein kinase B/Twist-related protein 1) signaling to sustain the cancer-associated fibroblasts features, thus supporting tumor cells growth and invasion.

Several studies in other types of cancers have shown involvement of MARCKS tumor expression in cancer progression, chemoresistance, and suggested that MARCKS inhibition could be a novel therapeutic approach. In breast cancer, MARCKS was reported to be implicated in tamoxifen-resistant MCF7 (Michigan Cancer Foundation-7) breast cancer cells and its inhibition decreased cell motility. The same study also noted correlations with poor-prognosis features and short OS [29]. More frequent IHC staining for phospho-MARCKS was found in breast cancer than in normal breast tissue and correlated with unfavorable prognostic parameters and metastatic status. Using both in vitro and in vivo models of breast cancer, the p-MARCKS was involved in resistance to paclitaxel treatment, with an increased paclitaxel sensitivity after reduction of p-MARCKS by knockdown or by treatment with MANS (p-MARCKS inhibitor peptide targeting the N-terminal myristoylation site) [30]. In a prior study, we showed that overexpression of MARCKS was correlated with the inflammatory breast cancer (IBC) phenotype. MARCKS stromal overexpression was more frequent in IBC than in non-IBC, which could sustain more cancer-associated fibroblasts activation in IBC and higher metastatic potential. We also showed the unfavorable prognostic value of MARCKS expression for metastasis-free survival in uni and multivariate analyses [31]. MARCKS was found to have an important role in the progression of colorectal cancer and to be implicated in cell motility, invasion and proliferation of colon cancer cells, whereas its inhibition clearly affected these cancer features and reduced the metastatic events [23]. The metastatic potential of p-MARCKS was shown in melanoma [21]. In prostate cancer, MARCKS promoted migration and invasion [32]. In cholangiocarcinoma, MARCKS overexpression correlated with poor survival, and experimental models showed the role of MARCKS in the migration of cholangiocarcinoma cells [24]. In a series of 99 patients with squamous cell lung carcinoma, a correlation was found between protein expression and poor survival [22]. In another study [28], MARCKS, specifically its phosphorylated form, was a key player in potentiating lung cancer cell migration/metastasis, suggesting a potential use of MARCKS-inhibition peptides in the treatment of lung cancer metastasis. Overexpression of p-MARCKS was associated with unfavorable survival in a series of 195 operated lung cancers and targeting MARCKS phosphorylation site domain (PSD) with MPS peptide (MARCKS phosphorylation site domain) suppressed tumor growth, metastasis and increased the sensibility to erlotinib treatment in vivo and in vitro [27]. A recent study demonstrated that a MARCKS ED peptide inhibited MARCKS phosphorylation, leading to an increase in sensitivity to radiation therapy [33]. Finally, in kidney cancer, MARCKS inhibition with MPS peptide synergistically interacted with regorafenib treatment and decreased survival of kidney cancer cells through inactivation of AKT and mTOR (mechanistic target of rapamycin) [34]. Thus, our observation of association of stromal MARCKS expression in EOC with shorter OS is consistent with the clinical and pre-clinical findings published in ovarian cancer and many other cancers.

## 4. Materials and Methods

### 4.1. Patients and Samples

We retrospectively collected pre-chemotherapy diagnostic tumor samples from 118 patients with epithelial ovarian cancer (EOC) treated between 2009 and 2015 at Institute Salah Azaiez of Tunis, Tunisia. These samples represented the operative specimen for women immediately operated or the surgical biopsies for women with non-operable disease thus treated with neo-adjuvant chemotherapy. Main inclusion criteria were pathologically-confirmed EOC, with available formaldehyde-fixed and paraffin-embedded pre-therapeutic tumor samples, available clinicopathological data, and signed informed patient’s consent. The control group included 40 normal ovarian tissues from Tunisian women operated for non-tumor ovarian lesions. All samples were spotted onto a tissue microarray before IHC analysis. The study was approved by our institutional ethics committee at Institut Salah Azaiez (N° 1646; 15 September 2016).

### 4.2. Tissue Microarray Construction

One tissue microarray (TMA) was constructed for all 158 cases, as previously described, with slight modifications [35]. For each sample, two representative tumor areas were carefully selected from a hematoxylin-eosin stained section of the donor block. Core cylinders with a diameter of 0.6 mm each were punched from each of these areas and deposited into the recipient paraffin block using a specific arraying device (Alphelys, Plaisir, France). Four-μm sections were made from the resulting TMA block, then used for IHC.

### 4.3. Western Blot Analyses

Before IHC analysis, we validated our MARCKS antibody by using western blot analysis with breast cancer cell lines. Expression was analyzed in three breast cancer cell lines (T47D, SUM149, MDA-MB-231) previously profiled using Affymetrix (Thermo Fisher Scientific, Inc., Rockford, IL, USA) DNA microarrays and for which MARCKS mRNA expression was documented as very low (T47D), moderate (SUM149), and very high (MDA-MB-231). Cells were washed 3 times with ice-cold PBS and then resuspended for 30 min in 750 μL of ice cold lysis buffer containing 50 mM Hepes, pH 7.5, 150 mM NaCl, 1.5 mM MgCl_2_, 1 mM EGTA, 1% Triton X-100, and 10% glycerol. A protease inhibitor mixture (Pierce, Thermo Fisher Scientific, Inc., Rockford, IL, USA) and the phosphotyrosyl phosphatase inhibitor sodium orthovanadate (BioLabs Cambridge, MA, USA) were added as recommended. Lysates were heated in SDS sample buffer (60 mM Tris-HCl, pH 6.7, 3% SDS, 2% (*v*/*v*) 2-mercaptoethanol, and 5% glycerol), separated by 10% SDS-PAGE, and transferred to nitrocellulose blotting membrane (Amersham, GE Healthcare Bio-Sciences, Pittsburgh, PA, USA). Membranes were blocked in PBS supplemented with BSA 5% and tween 0.1% for 1h30 and then incubated overnight at 4 °C with indicated antibodies. Visualization was done with ECL (Pierce, Thermo Fisher Scientific, Inc., Rockford, IL, USA).

### 4.4. Immunohistochemistry Analysis

MARCKS protein expression was analyzed on TMA for all 118 EOC and 40 normal ovarian tissues using standard IHC protocols. Paraffin sections were pretreated in PH6 PT Link (Agilent, Santa Clara, CA, USA). We used the rabbit monoclonal antibody, anti-MARCKS (D88D11) XP^®^Rabbit mAb#5607, from Cell Signaling Technology (dilutionat1/400) for staining and the Flex system (Dako, Agilent, Santa Clara, CA, USA) using a peroxidase enzyme for antigen revelation. Sections counterstained with hematoxylin were independently evaluated by two experienced ovarian pathologists (RD and MD) using light microscopy. Immunostaining scoring of epithelial cancer cells and of stromal cells was evaluated on the basis of staining intensity and positively stained areas as previously described [31]. MARCKS-negative cases were defined by 0% level expression and positive cases by at least 1% stained cells.

### 4.5. Statistical Analysis

Data were summarized by numbers and percentages for categorical variables, and median and range for continuous variables. Correlations between tumor groups and clinicopathological features were analyzed using the t-test or the Fisher’s exact test when appropriate. Follow-up was calculated from the date of diagnosis to the date of last news for event-free patients. Overall survival (OS) was calculated from the date of diagnosis until the date of death from any cause. Survival was calculated using the Kaplan-Meier method and curves were compared with the log-rank test. Uni- and multivariate prognostic analyses for OS were performed using Cox regression analysis (Wald test). Variables with a *p*-value ≤ 0.05 in univariate analysis were tested in multivariate analysis. All statistical tests were two-sided at the 5% level of significance. Analyses were done using the survival package (version 2.30) in the R software (version 2.9.1; http://www.cran.r-project.org/). We followed the reporting REcommendations for tumor MARKer prognostic studies (REMARK criteria) [36].

## 5. Conclusions

We showed MARCKS protein overexpression in the stroma of EOC and its association with shorter OS. The strengths of our study include the number of cases tested with a total of 118 EOC; to our knowledge, it is the first study analyzing specifically MARCKS protein expression in a large series of EOC. Importantly, we highlighted the association of MARCKS protein expression with poor OS in uni- and multivariate analyses, whereas the only other published study in ovarian cancer showed the association of MARCKS overexpression with poor OS only at the mRNA level and without multivariate analysis. Limitations of our study include its retrospective nature and associated biases such as missing data with the absence of survival information and/or of information about important prognostic factors such as macroscopic residual disease after surgery for all patients. Our results suggest that MARCKS overexpression in stromal cells of EOC might contribute to cancer-associated fibroblasts activation and contribute to the poor prognosis of disease. These findings suggest that inhibition of MARCKS could be a new potential therapeutic approach in EOC.

## Figures and Tables

**Table 1:** Clinicopathological characteristics of patients.

Characteristics		*N* (%)
Age		55 (27–85)
Pathological type	clear cell	5 (4%)
	endometrioid	3 (3%)
	mixed	4 (3%)
	serous	106 (90%)
FIGO Stage	1–2	10 (8%)
	3–4	108 (92%)
Pathological grade	1	5 (4%)
	2–3	113 (96%)
Surgery	no	0 (0%)
	yes	118 (100%)
Adjuvant and/or neo-adjuvant chemotherapy	no	12 (12%)
	yes	89 (88%)
Macroscopic residual disease after surgery	no	47 (46%)
	yes	55 (54%)
Deaths *		15 (22%)
5-year OS *, [CI95]		35% (22–56)
Median OS, months (range) *		44 (1–172)

* concerns the 68 patients treated with surgery and carboplatin-paclitaxel chemotherapy and with available follow-up. FIGO: Fédération Internationale de Gynécologie et d Obstétrique; OS: overall survival.

**Table 2:** Clinicopathological correlations with MARCKS expression (epithelial and stromal).

Characteristics	Epithelial MARCKS IHC Status				Stromal MARCKS IHC Status			
	*N*	Negative (*N* = 59)	Positive (*N* = 59)	*p*-Value	*N*	Negative (*N* = 27)	Positive (*N* = 91)	*p*-Value
Age	118	54 (30–85)	57 (27–79)	0.85	118	57 (27–85)	55 (30–80)	0.171
Pathological type				1				0.847
clear cell	5	2 (3%)	3 (5%)		5	1 (4%)	4 (4%)	
endometrioid	3	2 (3%)	1 (2%)		3	0 (0%)	3 (3%)	
mixed	4	2 (3%)	2 (3%)		4	0 (0%)	4 (4%)	
serous	106	53 (90%)	53 (90%)		106	26 (96%)	80 (88%)	
FIGO Stage				0.743				0.694
1–2	10	6 (10%)	4 (7%)		10	3 (11%)	7 (8%)	
3–4	108	53 (90%)	55 (93%)		108	24 (89%)	84 (92%)	
Pathological grade				0.226				0.159
1–2	35	21 (36%)	14 (24%)		35	11 (41%)	24 (26%)	
3	83	38 (64%)	45 (76%)		83	16 (59%)	67 (74%)	
Macroscopic residual disease after surgery				0.321				0.213
no	47	21 (40%)	26 (52%)		47	12 (60%)	35 (43%)	
yes	55	31 (60%)	24 (48%)		55	8 (40%)	47 (57%)	
Epithelial MARCKS IHC								0.662
negative					59	15 (56%)	44 (48%)	
positive					59	12 (44%)	47 (52%)	
Stromal MARCKS IHC				0.662				
negative	27	15 (25%)	12 (20%)					
positive	91	44 (75%)	47 (80%)					

**Table 3:** Uni- and multivariate analyses for OS.

Characteristics		Univariate			Multivariate		
		N	HR [95CI]	*p*-Value	N	HR [95CI]	*p*-Value
Age		68	1.02 [0.99–1.05]	0.304			
Pathological type	endometrioid versus clear cell	68	8.61 [0.74–100]	0.132			
	mixed versus clear cell		3.00 [0.27–33.7]				
	serous versus clear cell		1.60 [0.22–11.9]				
FIGO Stage	3–4 versus 1–2	68	4.18 [0.54–32.1]	0.169			
Pathological grade	3 versus 1–2	68	2.51 [1.14–5.49]	2.16 × 10^−2^	68	2.21 [1.01–4.86]	4.82 × 10^−2^
Macroscopic residual disease after surgery	yes versus no	65	1.57 [0.77–3.20]	0.212			
Epithelial MARCKS IHC	positive versus negative	68	0.95 [0.45–1.97]	0.881			
Stromal MARCKS IHC	positive versus negative	68	2.59 [1.06–6.36]	3.77 × 10^−2^	68	2.24 [0.91–5.53]	7.90 × 10^−2^

## Supplementary Materials

The following are available online at www.mdpi.com/1422-0067/19/1/41/s1.

## References

[1] Bowtell D.D. (2010). The genesis and evolution of high-grade serous ovarian cancer. Nat. Rev. Cancer.

[2] Ferlay J., Soerjomataram I., Dikshit R., Eser S., Mathers C., Rebelo M., Parkin D.M., Forman D., Bray F. (2015). Cancer incidence and mortality worldwide: Sources, methods and major patterns in GLOBOCAN 2012. Int. J. Cancer.

[3] Vang R., Shih I.-M., Kurman R.J. (2009). Ovarian low-grade and high-grade serous carcinoma: Pathogenesis, clinicopathologic and molecular biologic features, and diagnostic problems. Adv. Anat. Pathol..

[4] Shih I.-M., Kurman R.J. (2004). Ovarian tumorigenesis: A proposed model based on morphological and molecular genetic analysis. Am. J. Pathol..

[5] Ledermann J.A., Raja F.A., Fotopoulou C., Gonzalez-Martin A., Colombo N., Sessa C. (2013). ESMO Guidelines Working Group Newly diagnosed and relapsed epithelial ovarian carcinoma: ESMO Clinical Practice Guidelines for diagnosis, treatment and follow-up. Ann. Oncol..

[6] Tothill R.W., Tinker A.V., George J., Brown R., Fox S.B., Lade S., Johnson D.S., Trivett M.K., Etemadmoghadam D., Locandro B. (2008). Novel molecular subtypes of serous and endometrioid ovarian cancer linked to clinical outcome. Clin. Cancer Res..

[7] National Comprehensive Cancer Network (2015). NCCN Clinical Practice Guidelines in Oncology, Ovarian Cancer Including Fallopian Tube Cancer and Primary Peritoneal Cancer.

[8] Liao J., Qian F., Tchabo N., Mhawech-Fauceglia P., Beck A., Qian Z., Wang X., Huss W.J., Lele S.B., Morrison C.D. (2014). Ovarian cancer spheroid cells with stem cell-like properties contribute to tumor generation, metastasis and chemotherapy resistance through hypoxia-resistant metabolism. PLoS ONE.

[9] Stark D., Nankivell M., Pujade-Lauraine E., Kristensen G., Elit L., Stockler M., Hilpert F., Cervantes A., Brown J., Lanceley A. (2013). Standard chemotherapy with or without bevacizumab in advanced ovarian cancer: Quality-of-life outcomes from the International Collaboration on Ovarian Neoplasms (ICON7) phase 3 randomised trial. Lancet Oncol..

[10] Ben Fatma L., Hochlef M., Gharbi O., Landolsi A., Limam S., Chabchoub I., Cherif N., Bouguizene S., Yacoubi T., Bibi M. (2006). Epithelial advanced ovarian carcinoma in the central region of Tunisia: Therapeutic results and prognostic factors on 104 patients. Bull. Cancer.

[11] Arai S., Fanale M., DeVos S., Engert A., Illidge T., Borchmann P., Younes A., Morschhauser F., McMillan A., Horning S.J. (2013). Defining a Hodgkin lymphoma population for novel therapeutics after relapse from autologous hematopoietic cell transplant. Leuk. Lymphoma.

[12] Sasse S., Klimm B., Görgen H., Fuchs M., Heyden-Honerkamp A., Lohri A., Koch O., Wilhelm M., Trenn G., Finke J. (2012). German Hodgkin Study Group (GHSG) Comparing long-term toxicity and efficacy of combined modality treatment including extended- or involved-field radiotherapy in early-stage Hodgkin’s lymphoma. Ann. Oncol..

[13] Behringer K., Mueller H., Goergen H., Thielen I., Eibl A.D., Stumpf V., Wessels C., Wiehlpütz M., Rosenbrock J., Halbsguth T. (2013). Gonadal function and fertility in survivors after Hodgkin lymphoma treatment within the German Hodgkin Study Group HD13 to HD15 trials. J. Clin. Oncol..

[14] Hanly P., Soerjomataram I., Sharp L. (2015). Measuring the societal burden of cancer: The cost of lost productivity due to premature cancer-related mortality in Europe. Int. J. Cancer.

[15] Ramsden J.J. (2000). MARCKS: A case of molecular exaptation?. Int. J. Biochem. Cell Biol..

[16] Singer M., Martin L.D., Vargaftig B.B., Park J., Gruber A.D., Li Y., Adler K.B. (2004). A MARCKS-related peptide blocks mucus hypersecretion in a mouse model of asthma. Nat. Med..

[17] Ouimet C.C., Wang J.K., Walaas S.I., Albert K.A., Greengard P. (1990). Localization of the MARCKS (87 kDa) protein, a major specific substrate for protein kinase C, in rat brain. J. Neurosci..

[18] Li Y., Martin L.D., Spizz G., Adler K.B. (2001). MARCKS protein is a key molecule regulating mucin secretion by human airway epithelial cells in vitro. J. Biol. Chem..

[19] Allen L.H., Aderem A. (1995). A role for MARCKS, the alpha isozyme of protein kinase C and myosin I in zymosan phagocytosis by macrophages. J. Exp. Med..

[20] Blackshear P.J. (1993). The MARCKS family of cellular protein kinase C substrates. J. Biol. Chem..

[21] Chen X., Rotenberg S.A. (2010). PhosphoMARCKS drives motility of mouse melanoma cells. Cell. Signal..

[22] Hanada S., Kakehashi A., Nishiyama N., Wei M., Yamano S., Chung K., Komatsu H., Inoue H., Suehiro S., Wanibuchi H. (2013). Myristoylated alanine-rich C-kinase substrate as a prognostic biomarker in human primary lung squamous cell carcinoma. Cancer Biomark..

[23] Rombouts K., Carloni V., Mello T., Omenetti S., Galastri S., Madiai S., Galli A., Pinzani M. (2013). Myristoylated Alanine-Rich protein Kinase C Substrate (MARCKS) expression modulates the metastatic phenotype in human and murine colon carcinoma in vitro and in vivo. Cancer Lett..

[24] Techasen A., Loilome W., Namwat N., Takahashi E., Sugihara E., Puapairoj A., Miwa M., Saya H., Yongvanit P. (2010). Myristoylated alanine-rich C kinase substrate phosphorylation promotes cholangiocarcinoma cell migration and metastasis via the protein kinase C-dependent pathway. Cancer Sci..

[25] Yang Y., Chen Y., Saha M.N., Chen J., Evans K., Qiu L., Reece D., Chen G.A., Chang H. (2015). Targeting phospho-MARCKS overcomes drug-resistance and induces antitumor activity in preclinical models of multiple myeloma. Leukemia.

[26] Yang Z., Xu S., Jin P., Yang X., Li X., Wan D., Zhang T., Long S., Wei X., Chen G. (2016). MARCKS contributes to stromal cancer-associated fibroblast activation and facilitates ovarian cancer metastasis. Oncotarget.

[27] Chen C.-H., Statt S., Chiu C.-L., Thai P., Arif M., Adler K.B., Wu R. (2014). Targeting myristoylated alanine-rich C kinase substrate phosphorylation site domain in lung cancer. Mechanisms and therapeutic implications. Am. J. Respir. Crit. Care Med..

[28] Chen C.-H., Thai P., Yoneda K., Adler K.B., Yang P.-C., Wu R. (2014). A peptide that inhibits function of Myristoylated Alanine-Rich C Kinase Substrate (MARCKS) reduces lung cancer metastasis. Oncogene.

[29] Browne B.C., Hochgräfe F., Wu J., Millar E.K.A., Barraclough J., Stone A., McCloy R.A., Lee C.S., Roberts C., Ali N.A. (2013). Global characterization of signalling networks associated with tamoxifen resistance in breast cancer. FEBS J..

[30] Chen C.-H., Cheng C.-T., Yuan Y., Zhai J., Arif M., Fong L.W.R., Wu R., Ann D.K. (2015). Elevated MARCKS phosphorylation contributes to unresponsiveness of breast cancer to paclitaxel treatment. Oncotarget.

[31] Manai M., Thomassin-Piana J., Gamoudi A., Finetti P., Lopez M., Eghozzi R., Ayadi S., Lamine O.B., Manai M., Rahal K. (2017). MARCKS protein overexpression in inflammatory breast cancer. Oncotarget.

[32] Dorris E., O’Neill A., Hanrahan K., Treacy A., Watson R.W. (2017). MARCKS promotes invasion and is associated with biochemical recurrence in prostate cancer. Oncotarget.

[33] Rohrbach T.D., Jones R.B., Hicks P.H., Weaver A.N., Cooper T.S., Eustace N.J., Yang E.S., Jarboe J.S., Anderson J.C., Willey C.D. (2017). MARCKS phosphorylation is modulated by a peptide mimetic of MARCKS effector domain leading to increased radiation sensitivity in lung cancer cell lines. Oncol. Lett..

[34] Chen C.-H., Fong L.W.R., Yu E., Wu R., Trott J.F., Weiss R.H. (2017). Upregulation of MARCKS in kidney cancer and its potential as a therapeutic target. Oncogene.

[35] Ginestier C., Charafe-Jauffret E., Bertucci F., Eisinger F., Geneix J., Bechlian D., Conte N., Adélaïde J., Toiron Y., Nguyen C. (2002). Distinct and complementary information provided by use of tissue and DNA microarrays in the study of breast tumor markers. Am. J. Pathol..

[36] McShane L.M., Altman D.G., Sauerbrei W., Taube S.E., Gion M., Clark G.M. (2005). Statistics Subcommittee of the NCI-EORTC Working Group on Cancer Diagnostics REporting recommendations for tumour MARKer prognostic studies (REMARK). Br. J. Cancer.

