# Supplementary material for: Stromal Expression of MARCKS Protein in Ovarian Carcinomas Has Unfavorable Prognostic Value
Source: Int J Mol Sci. 2017 Dec 23;19(1):41. doi: 10.3390/ijms19010041 (PMC5795991; doi:10.3390/ijms19010041)
Supplement: Supplementary file 1 [file ijms-19-00041-s001.pdf]

Suppl Figure S1

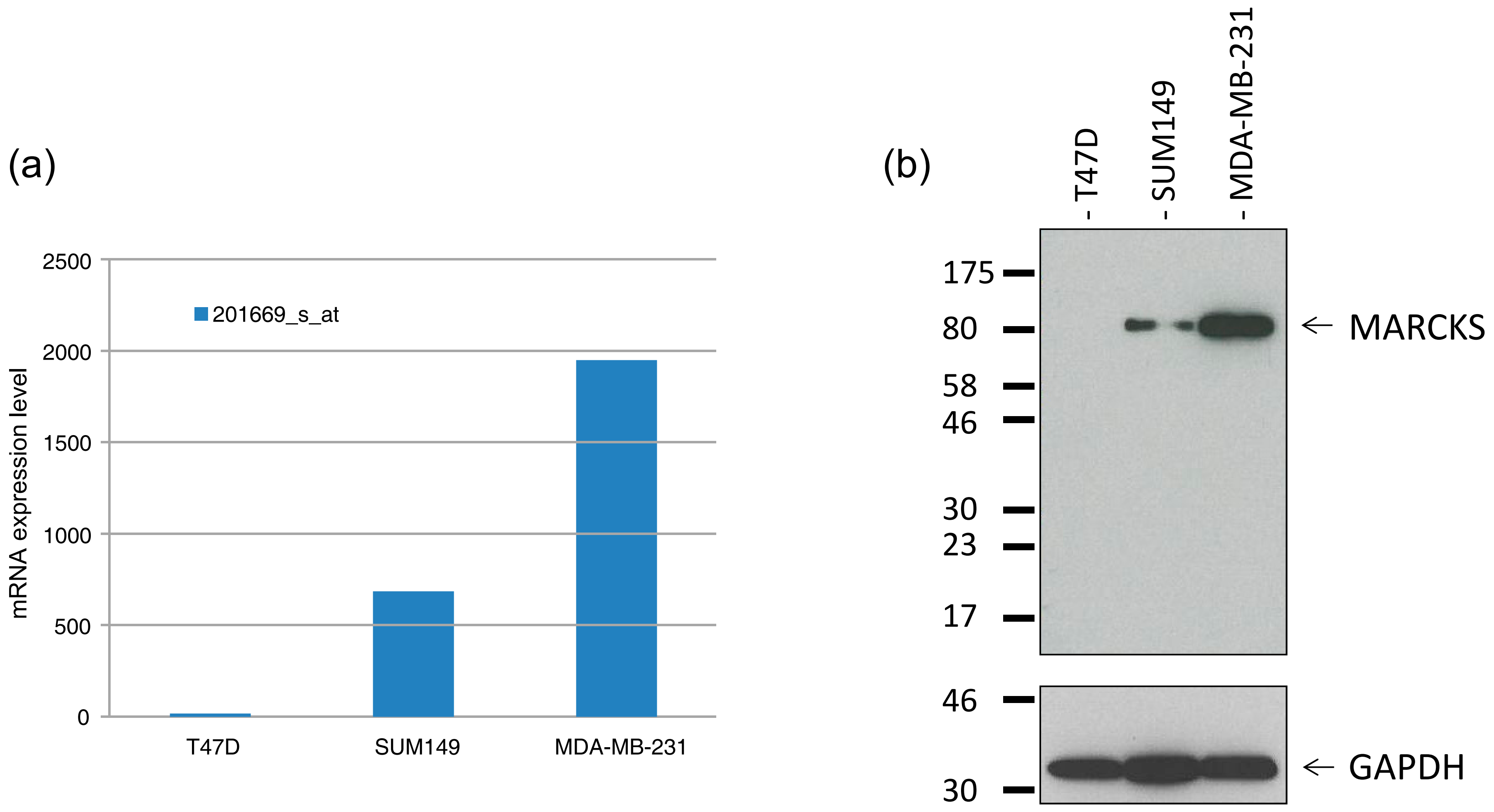

Supplementary Figure S1: MARCKS mRNA and protein expression in breast cancer cell lines
(a) mRNA expression level measured using Affymetrix DNA microarrays in three breast cancer cell lines with different expression levels.
(b) Western blot analysis using the anti-MARCKS (D88D11) XP® Rabbit monoclonal Ab #5607 from Cell Signaling Technology in the same cell lines (total lysate). The antibody recognizes MARCKS (only one band at the right size) and a good correlation is observed between MARCKS mRNA and protein expression levels.

Suppl Figure S2

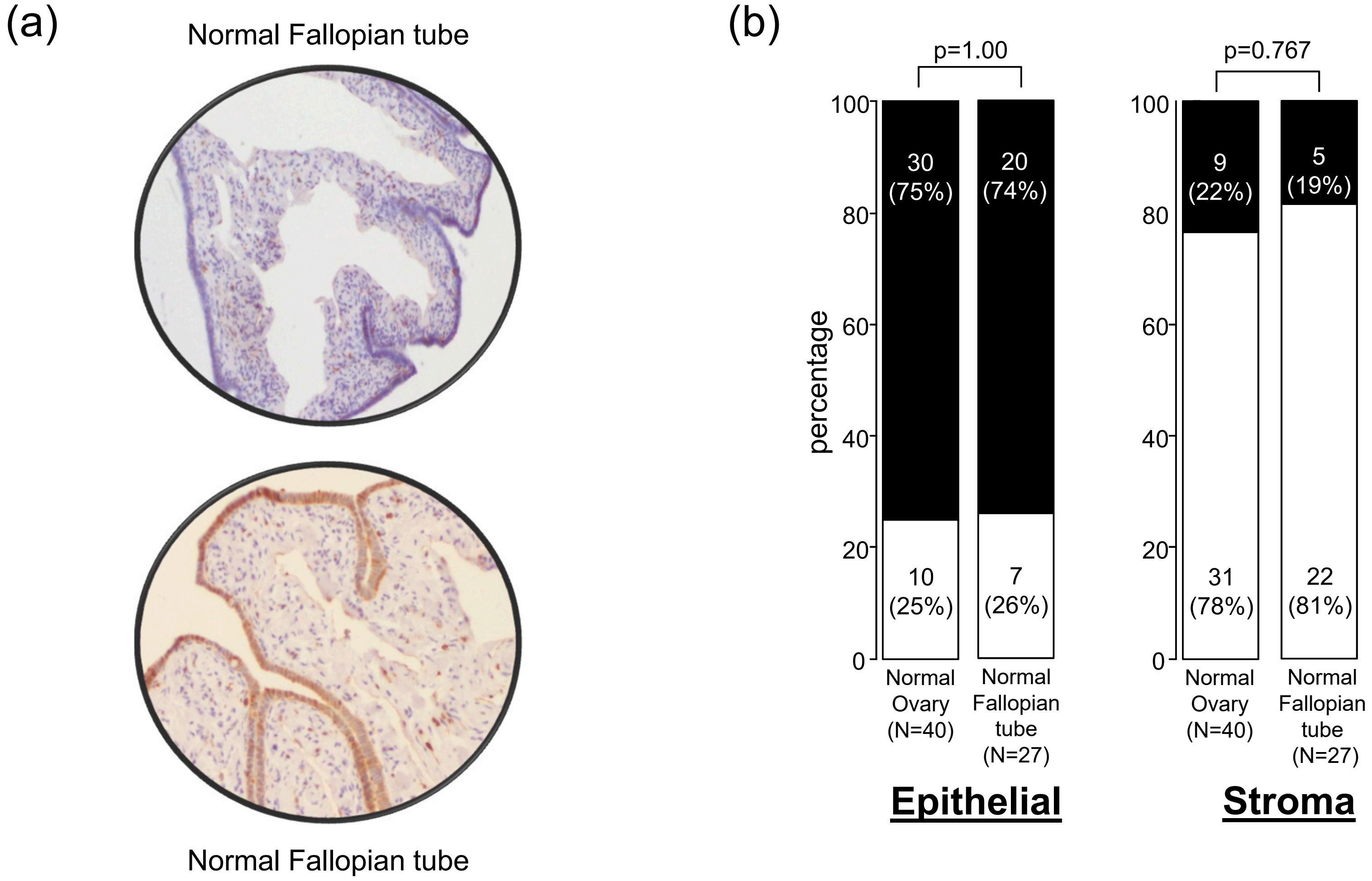

Supplementary Figure S2: MARCKS immunostaining in normal Fallopian tube.
(a) Representative images of normal Fallopian tube with weak expression of MARCKS in both stromal and epithelial cells (top) and with weak expression in stromal cells but strong in epithelial cells (bottom). **(b)** Box plots showing the percentage of MARCKS positive (black) samples and MARCKS negative (white) samples between normal ovary and Fallopian cells for the epithelial (left) and stromal (right) staining. The p-values are for the Fisher's exact test.
